# Biobanking of different body fluids within the frame of IVF—a standard operating procedure to improve reproductive biology research

**DOI:** 10.1007/s10815-016-0847-5

**Published:** 2016-11-26

**Authors:** Michael Schenk, Berthold Huppertz, Barbara Obermayer-Pietsch, Darja Kastelic, Martina Hörmann-Kröpfl, Gregor Weiss

**Affiliations:** 1 Das Kinderwunsch Institut Schenk GmbH, Am Sendergrund 11, 8143 Dobl, Austria; 2 Biobank Graz, Medical University of Graz, Neue Stiftingtalstrasse 2, 8010 Graz, Austria; 3 Division of Endocrinology and Diabetology, Department of Internal Medicine, Medical University of Graz, Auenbruggerplatz 15, 8036 Graz, Austria

**Keywords:** Biobanking, IVF, Follicular fluid, Steiner-TAN needle, Standard operating procedure

## Abstract

**Purpose:**

The aim of the present study was to develop a standard operating procedure (SOP) for the collection, transport, and storage of human cumulus cells, follicular fluid, blood serum, seminal plasma, embryo culture supernatant, and embryo culture supernatant control obtained within the IVF process under approved protocols and written informed consent from participating patients. The SOP was developed at the Kinderwunsch Institut Schenk, Dobl, Austria, together with Biobank Graz of the Medical University of Graz, Austria.

**Methods:**

The SOP provides comprehensive details of laboratory procedures and sampling of the different fluids within the IVF process. Furthermore, information on sample coding, references of involved laboratory techniques (e.g., oocyte retrieval with a Steiner-TAN needle), ethical approvals, and biobanking procedures are presented.

**Results:**

The result of the present study is a standard operating procedure.

**Conclusions:**

The SOP ensures a professional way for collection and scientific use of IVF samples by the Kinderwunsch Institut Schenk, Dobl, Austria, and Biobank Graz of the Medical University of Graz, Austria. It can be used as a template for other institutions to unify specimen collection procedures in the field of reproductive health research.

## Introduction

Infertility is a global phenomenon, affecting an estimated 48.5 million of reproductive-aged couples worldwide in 2010. The overall burden of infertility has remained similar in estimated levels and trends according to a WHO study published in 2012 [1]. This persisting problem of infertility explains the ever increasing number of people receiving care in terms of in vitro fertilization (IVF), the assisted reproductive technique (ART), which is now existing for almost 40 years [2]. What is known so far is that success in the IVF treatment depends on a complex interplay of reproductive medicine and clinical embryology. Changes in stimulation protocols were thought to optimize the therapeutic outcome; however, clinical pregnancy rates or baby take home rates did not increase as expected [3]. Improving the process of IVF by different research approaches is tightly linked to state-of-the-art methods of sampling, transporting, and storing of biological materials.

In terms of storage, biobanks have become indispensable institutions for archiving biological materials. In their professional capacity, they do not only provide a vast amount of different sample types, but are also in the ascendant to act as research partners for upcoming scientific questions. Biobanking studies in the field of reproductive medicine are still a niche in IVF research, with only a few publications so far [4, 5]. This unused scientific potential is possibly caused by additional ethical hurdles concerning handling of embryonic specimens, cells, fluids, and tissues in terms of processing and storage (i.e., cord blood, oocytes, etc.).

The mechanisms of collecting the samples are crucial for the overall usefulness of the specimens for scientific research and clinical purpose. Thus, standard operating procedures (SOP) are required to standardize the different collections and to facilitate comparability of international institutions of reproductive medicine.

Here, we describe an SOP for collecting, processing, handling, and storing samples derived from patients undergoing IVF treatment comprising cumulus cells (CC), follicular fluid (FF), blood serum (SR), seminal plasma (SE), embryo culture supernatant (SU), and respective culture control media (SUC). These fluids are promising candidates to investigate and correlate a variety of different parameters within the process of IVF.

A major advantage of the present SOP is the fact that follicular fluid is collected with the Steiner-TAN-Needle, which allows the collection of follicular fluid per oocyte instead of pooling all the follicular fluids [6]. This technique allows linking of one particular oocyte to the respective IVF outcome. Together with modern IVF techniques like time-lapse culture systems [7] and preimplantation genetic screening (PGS) [8], this SOP provides an efficient and powerful method, addressing a broad range of applications for different scientific questions and IVF studies.

In the recent years, follicular fluid (FF) has become a major target for oocyte quality in human IVF. To limit embryo “overproduction,” there is a trend to start the selection process with the oocytes rather than with the already-fertilized oocytes [9]. The follicular fluid comprises a variety of different proteins including anti-apoptotic proteins and metalloproteinase- and IGF-related proteins as well as other growth factors like angiotensinogen (AGT), growth hormone receptor (GHR), or hepatocyte growth factor-like protein (MST1) [10], thus serving as potential target for scientific questions. Besides blood serum, seminal, and embryo culture supernatant, cumulus cells have evolved to become interesting targets as predictors of blastocyst formation and pregnancy success overall [11].

## Methods and results

The SOP was established by the Kinderwunsch Institut Schenk, Dobl, Austria, together with Biobank Graz [12] of the Medical University of Graz, Austria, to facilitate future international collaborations and to possibly improve the process of IVF by gaining more insights into molecular mechanisms and processes in early stages of embryogenesis. In the different sections of the SOP, we outline the procedure from responsibilities, via documents and references, to coding and sampling of the different fluids. Furthermore, we essentially included all of the materials and the respective companies used and list the sampling processes in detail. Below, we refer to details of the different SOP sections.

### Purpose, scientific value, responsibilities, references, abbreviations, materials, and sample collection approvals

Section 1 presents the purpose of the SOP, including information on collected materials and how it was developed. Sections 2 and 3 state the scientific value and the scope. Section 4 comments on the responsibilities of the involved directors/principal investigators and authorized staff members. In section 5, references and documents are highlighted which contributed to the preparation of the SOP. A list of all used abbreviations is found in section 6. Section 7 reveals a list of materials, instruments, and reagents with respective companies, for an accurate implementation of the SOP. The section is divided in general equipment, instruments, microscopes and related equipment, and culture media and embryo culture equipment as well as tubes, dishes, and tips. Section 8 shows the approval list for sampling, collecting, and storage of bio-specimens, including ethical and patient approvals.

### Coding, sample preparation, and storage and use

**Fig. 1:**
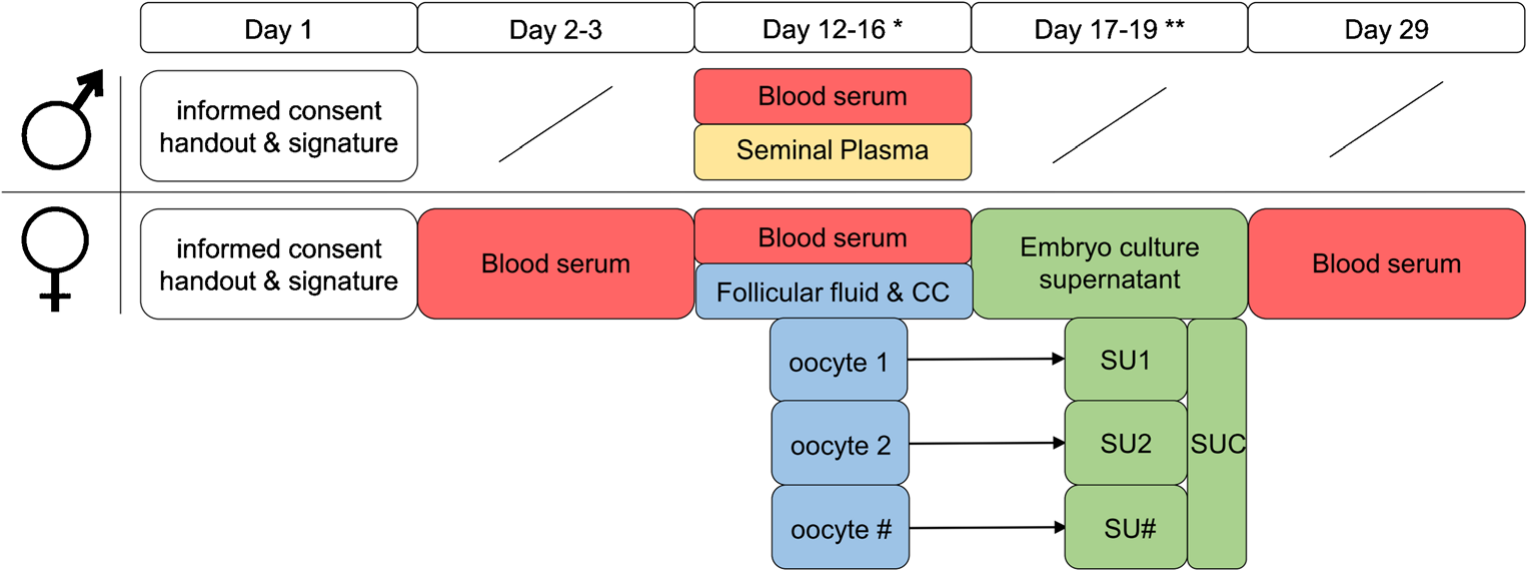
Schedule of sampling different fluids within the IVF process. The graph shows the timeline of sampling blood serum (SR), seminal plasma (SE), follicular fluid (FF), cumulus cells (CC), embryo culture supernatant (SU), and control media (SUC). The *number sign* (#) stands for a varying number of oocytes collected during the IVF process. *Asterisk* indicates day of oocyte retrieval; *double asterisks* indicate day of embryo transfer and/or embryo cryoconservation

The coding system of the Kinderwunsch Institut Schenk is shown in section 9. It serves as paradigm for other institutions, including relevant information of the patient, type of sample, and collection ID. Even if other coding systems are used, it is suggested to include the essential information on the samples and make sure that the sample type can be tracked and linked to the appropriate patient record. In section 10, we provide detailed protocols of sampling of blood serum, follicular fluid, cumulus cells, seminal plasma, embryo culture supernatant, and supernatant control. The section provides information on authorities of performing the procedure, labeling of samples, and storage at the biobank. Furthermore, the specific days of sampling are provided in the SOP and in Fig. 1. Section 11 provides the sample usage with different methods of analyzing proteomics and metabolomics.

During the whole procedure of the SOP, one has to keep in mind that every sample has to be considered as potentially infectious. Thus, wearing protective latex gloves is mandatory when working with biological specimens at all time according to the Level 2 criteria of the European Biosafety Association (http://www.ebsaweb.eu/). It is not advisable to handle more than one sample at the same time to avoid any possibility of confusion and/or contamination. It is also necessary to use separate sets of sterile instruments to avoid cross-contaminations. Each institution may have their own regulations for both transporting and handling human samples under Biosafety Level 2 protocols (BSL2). However, the safety standards in the respective institution should be ensured and followed. If institutions do not have the ability to store their samples in a biobank, it is recommended to store samples at −80 °C for long-term storage.

1. Purpose
  The present standard operating procedure (SOP) describes the process of collecting, transporting, and storing human body fluid samples (blood serum, cumulus cells, follicular fluid, seminal plasma, and embryo culture supernatants) at a biobank for research purposes. The SOP was developed at the Kinderwunsch Institut Schenk, Dobl, Austria, together with Biobank Graz of the Medical University of Graz, Austria.
2. Scientific value
  The storage of all samples enables retrospective, scientific research, leading to a better understanding of pathology and progression of diseases in the field of reproductive biology. Furthermore, it permits optimization of diagnostics and therapeutic approaches.
3. Scope
  This procedure applies to blood serum, cumulus cells, follicular fluid, seminal plasma, embryo culture supernatant, and supernatant control samples collected for research in the field of reproductive biology.
4. Responsibilities
  4.1 The Principal Investigator of the IVF institution and the Director of Biobank have to maintain the standard operating procedure.
  4.2 Every sample collection operator involved in the process of the SOP has to adhere to the procedure.
  4.3 The Principal Investigator of the IVF institution and the Director of Biobank have to ensure that the procedure is followed and that all personnel adhere to the procedure.
5. References and documents
  5.1 Rose BI, Laky D. A comparison of the Cook single lumen immature ovum IVM needle to the Steiner-Tan pseudo double lumen flushing needle for oocyte retrieval for IVM. J Assist Reprod Genet 2013;30(6):855–60.
  5.2 Steiner H-P. The effect of needle diameter on duration of oocyte collection procedure. Hum Reprod 2011; 26(12):3495–3495
  5.3 Sheldon E, Vo KC, McIntire RA, Aghajanova L, Zelenko Z, Irwin JC, et al. Biobanking human endometrial tissue and blood specimens: standard operating procedure and importance to reproductive biology research and diagnostic development. Fertil Steril 2011;95(6):2120–2122.e12.
  5.4 UCSF Guide for the Research Use of Human Biological Specimens: Collecting, Banking and Sharing Specimens. San Francisco: USCF, 2005.
6. Abbreviations
  CC: Cumulus cells
  FF: Follicular fluid
  FL: Fluid
  IC: Informed consent
  ID: Identification number
  IVF: *In vitro* fertilization
  KIWI: Kinderwunsch Institut Schenk
  SE: Seminal plasma
  SOP: Standard operating procedure
  SR: Blood serum
  SU: Supernatant
  SUC: Supernatant control
  QR: Quick response
7. Materials and reagents
  7.1 General equipment
    Adhesive label
    Label writer
    Medical records
    Protective latex gloves (biosafety level 2)
    Water-resistant pen
  7.2 Instruments
    Centrifuge EBA 20 (Andreas Hettich GmbH & Co.KG, Germany)
    Fridge Medline (Liebherr-International Deutschland GmbH, Germany)
    Incubator Forma (Thermo Fisher Scientific, USA)
    IVF workstation L24E with heating stage (K-SYSTEMS Kivex Biotec A/S, Denmark)
    Needle 21G (Terumo Deutschland GmbH, Germany)
    Pioneer Pro Pump (Pioneer Pump Limited, UK)
    Pipette (0.5–10 μl; 100–1000 μl) (Eppendorf AG, Germany)
    Steiner flush/valve (IVFETFLEX.com HandelsgmbH & Co KG, Austria)
    Steiner-Tan Needle 17 gauge (IVFETFLEX.com HandelsgmbH & Co KG, Austria)
    Sterile transfer pipette (VWR International GmbH, Austria)
    Syringe 1 ml (Terumo Deutschland GmbH, Germany)
    Syringe norm-ject disposable (60 ml) (Henke-Sass, Wolf GmbH, Germany)
    Tube warmer (Origio Clinical Monitoring GmbH, Germany)
  7.3 Microscope and equipment
    Cover glass 18 × 18 mm (Waldemar Knittel Glasbearbeitungs GmbH, Germany)
    Object slide 76 × 26 × 1 mm (Engelbrecht Medizin- und Labortechnik GmbH, Germany)
    Phase contrast microscope DMLS (×10, ×20, ×40 objective) (Leica Camera AG)
    Stereomicroscope Leica MZ95 (Leica Camera AG, Germany)
    Stereomicroscope Nikon SMZ1500 (Nikon GmbH, Austria)
  7.4 Culture media and embryo culture equipment
    Embryoslide (Vitrolife AB, Sweden)
    Flushing medium GM501 Flush (Gynemed Medizinprodukte GmbH & Co.KG, Germany)
    Hyaluronidase (Gynemed Medizinprodukte GmbH & Co.KG, Germany)
    Mineral oil (Gynemed Medizinprodukte GmbH & Co.KG, Germany)
    Sperm air medium (Gynemed Medizinprodukte GmbH & Co.KG, Germany)
    Universal culture medium (Gynemed Medizinprodukte GmbH & Co.KG, Germany)
  7.5 Tubes, dishes, and tips
    Centrifuge tube (15 ml) (VWR International GmbH, Austria) [cat#: 525–0400]
    Culture dish (five wells) (MTG Medical Technology Vertriebs-GmbH, Germany) [cat#: *3003177301]
    Eppendorf tips (0.1–10 μl) single packed (Eppendorf AG, Germany) [cat#:30010019]
    Eppendorf tubes (1.5 ml) (Eppendorf AG, Germany) [cat#:0030120086]
    IVF round dish 60 × 15 mm (VWR International GmbH, Austria) [cat#:353652]
    IVF round dish cell culture dish 35 × 10 mm (VWR International GmbH, Austria) [cat#:353801]
    Multipurpose beaker 100 ml sterile (Greiner Bio-One International GmbH, Germany) [cat#:724473]
    Pipette tips (0.5–10 μl; 100–1000 μl) (Eppendorf AG, Germany) [cat#:0030 000.919]
    Round bottom tube (15 ml) (VWR International GmbH, Austria) [cat#:35–2001]
    Vacuette serum tube (Greiner Bio-One International GmbH, Germany) [cat#:455071]
8. Sample collection approvals
  8.1 Requirements:
    8.1.1 Ethical review committee
      8.1.1.1 An authorization to work with human-derived samples is required.
      8.1.1.2 Apply for an ethic vote at your local ethical review committee.
      8.1.1.3 The ethical review committee will review the available protocols.
      8.1.1.4 Sampling cannot be realized without positive approval of the ethical committee.
    8.1.2 Patient(s)
      8.1.2.1 For the collection of follicular fluid, seminal plasma, blood serum, supernatant, and supernatant control, a positive-signed written informed consent (IC) from each participating patient is required.
      8.1.2.2 The document is to be handed out and explained to the patient prior to sampling.
      8.1.2.3 The medical doctor or authorized staff ensures that the patient thoroughly understands the informed consent before signing it.
      8.1.2.4 Staff from the patient management has to note the respective person and add the IC to the patient chart.
      8.1.2.5 A copy of the IC is handed over to the patient.
      8.1.2.6 Refer to supplemental material or Biobank Graz webpage for an example of informed consent (http://www.medunigraz.at/strategische-projekte/biobank/special-information-for-researchers/ethical-legal-and-social-issues/informed-consent-of-biobank-graz/).
    8.1.3 Medical data protection
      8.1.3.1 Patient’s medical data protection has to be assured at all time.
      8.1.3.2 The patient has to sign the informed consent.
      8.1.3.3 The medical doctor or authorized staff has to sign that he/she explained the IC to the patient.
      8.1.3.4 Every patient participating gets an identification number (anonymization).
      8.1.3.5 The identification number of the patient is deposited in the QR code of every sample (pseudonymization).
      8.1.3.6 From the moment, the samples are designated for biobanking and leave the institution; it is only the IVF facility which can backtrack the samples stored at a biobank.
      8.1.3.7 Linkage to patients’ medical records is assured by the IVF facility.
9. Coding
  For tracing reasons, every sample gets an adhesive label comprising a QR code and a human readable code. This code was developed at Biobank Graz and is generated during sample collection in an excel sheet with the TBarCode-Add-In (TEC IT) for Microsoft Office. This coding system is accomplished in the Kinderwunsch Institut Schenk, Dobl, Austria, and serves as a paradigm. It can be adjusted within different institutions. The barcode is built as follows:
  KIWI/123456/YYYYMMDD/FL/KI01/XXYQQ
  Meaning of the individual elements:
  KIWI: Biobank abbreviation for Kinderwunsch Institut Schenk
  123456: Six-digit KIWI-ID of the patient
  YYYY/MM/DD: Date of sampling in format YYYY/MM/DD (year, month, day)
  FL: Type of fluid (FL) sample (CC, cumulus cells; FF, follicular fluid; SR, blood serum; SE, seminal plasma; SU, supernatant; SUC, supernatant control)
  KI01: Collection ID (biobank), which grants association between sample and collection of one sender
  XX: double digit number, starting at 01 (01 = first sample of one fluid type of one patient at a certain date of sampling, 02 = second sample, etc.)
  Y: Samples with follicular fluid (FF): number of oocytes.
    All other samples = 0
  QQ: Samples with follicular fluid (FF): total number of all follicles punctured
10. Sample preparation
  10.1 Blood serum (SR)
    10.1.1 Preparation
      10.1.1.1 Blood collection is to be performed by a medical doctor or authorized staff.
      10.1.1.2 Print a label with name and sample ID of the patient.
      10.1.1.3 Inquire the full name and date of birth of the patient and compare with the label to assure patient’s identity.
      10.1.1.4 Note time of sampling and name of operating person.
      10.1.1.5 Collect at least 4 ml venous blood in an 8-ml vacuette tube (with serum separator).
      10.1.1.6 Store sample for 30 min at 37 °C.
      10.1.1.7 Use balance tubes and centrifuge samples at 1800*g* for 10 min at room temperature.
      10.1.1.8 Collect at least 3 ml supernatant (=blood serum, SR) in 15-ml tubes and store at 4 °C until transfer to biobank.
      10.1.1.9 Right before transport, put samples in a polystyrene rack and deliver the box to a haulage service.
      10.1.1.10 The haulage service assures a save transport to biobank under a consistent-refrigerated temperature (4 °C).
      10.1.1.11 Samples have to reach biobank within 24 h after sampling.
      10.1.1.12 Biobank assures validity of obtained SR samples by checking the respective barcode.
      10.1.1.13 Open SR samples manually and place into a Hamilton sample carrier.
      10.1.1.14 Fully automated Hamilton pipetting robot ML-STAR splits obtained serum samples into six aliquots of 0.5 ml each.
      10.1.1.15 Store SR samples at −80 °C in storage systems of biobank for long-term storage.
    10.1.2 Schedule of sampling
      10.1.2.1 Women
        10.1.2.1.1 Standard blood sampling (before hormone treatment; day 2–3)
        10.1.2.1.2 Day of oocyte retrieval (day 12–16)
        10.1.2.1.3 Day of pregnancy test (∼10 days after embryo transfer)
      10.1.2.2 Men
        10.1.2.2.1 Day of oocyte retrieval
  10.2 Follicular fluid (FF) and cumulus cells (CC)
    10.2.1 Preparation
      10.2.1.1 Follicular fluid collection is performed by a medical doctor or authorized staff.
      10.2.1.2 Day before oocyte retrieval
        10.2.1.2.1 Prepare 2 ml of flushing medium for every expected follicle and 2 ml of flushing medium to rinse the pump system in a 60 ml disposable syringe and store it at 37 °C overnight.
        10.2.1.2.2 Prepare a five-well culture dish with two drops (=2× 25 μl) of universal culture media per well and cover with 1 ml mineral oil.
        10.2.1.2.3 Mark the drops with numbers on top and bottom of the culture dish.
        10.2.1.2.4 Equilibrate the culture dish overnight at 37 °C, 5.5% CO_2_ in the incubator.
      10.2.1.3 Day of oocyte retrieval
        10.2.1.3.1 Prepare two 14-ml round tubes for every follicle expected.
        10.2.1.3.2 Prepare a five-well culture dish with warm flushing media (37 °C).
        10.2.1.3.3 Put the syringe with flushing medium in the Steiner flush/valve station right before puncture and rinse the pump system.
        10.2.1.3.4 Note time of sampling and name of operating person.
        10.2.1.3.5 Assure patient’s identity before oocyte retrieval.
        10.2.1.3.6 Puncture and flush every single follicle with the Steiner-Tan Needle 17 gauge.
        10.2.1.3.7 Collect follicular fluid and flushing medium separately in pre-warmed and labeled round bottom tubes (at least 2–5 ml per follicle).
        10.2.1.3.8 Put obtained fluids into sterile IVF round dishes 60 × 15 mm.
        10.2.1.3.9 Examine FF and flushed medium for oocytes with a stereo microscope on the heating stage of the IVF workstation.
        10.2.1.3.10 Culture single oocytes in labeled five-well culture dish with flushing media.
        10.2.1.3.11 Put FF back in a 14-ml round bottom tube.
        10.2.1.3.12 Transfer FF into a 15-ml centrifuge tube with a screw cap.
        10.2.1.3.13 Store tubes at 4 °C until transfer to biobank.
        10.2.1.3.14 Right before transport, put samples in a polystyrene rack and deliver the box to a haulage service.
        10.2.1.3.15 The haulage service assures a save transport to biobank under a consistent refrigerated temperature (4 °C).
        10.2.1.3.16 Samples have to reach biobank within 24 h after sampling.
        10.2.1.3.17 Biobank assures validity of obtained FF samples by checking the respective barcode.
        10.2.1.3.18 Centrifuge samples (20 min, 867 rpm), collect cumulus cells from the bottom of the tube and store at −80 °C.
        10.2.1.3.19 Collect supernatant (=FF) and place into a Hamilton sample carrier.
        10.2.1.3.20 Fully automated Hamilton pipetting robot ML-STAR splits obtained serum samples into four aliquots of 1 ml each.
        10.2.1.3.21 Store FF samples at −80 °C in storage systems of biobank for long-term storage.
      10.2.1.4 IVF preparation
        10.2.1.4.1 Perform IVF procedure with a stereo microscope on the heating stage of the IVF workstation.
        10.2.1.4.2 Take a dish cell culture dish 35 × 10 mm and put three drops hyaluronidase with three drops sperm air next to every hyaluronidase drop.
        10.2.1.4.3 Remove cumulus cells by gently up and down pipetting of the oocyte in the hyaluronidase (maximum 40 s).
        10.2.1.4.4 Every oocyte must be treated individually for accurate tracking of the respective oocyte.
        10.2.1.4.5 Wash oocyte by gently up and down pipetting of the oocyte in the three drops of sperm air.
        10.2.1.4.6 Transfer oocyte into five-well culture dish (prepared with universal culture media and mineral oil the day before).
        10.2.1.4.7 Perform IVF or ICSI 1–4 h after oocyte retrieval.
        10.2.1.4.8 Culture single embryo in 25-μl universal culture medium covered with mineral oil in embryo slides 3–5 days.
        10.2.1.4.9 Culture 25-μl universal culture medium in a separate well in the same embryo slide (without embryo) as embryo culture supernatant control (SUC) 3–5 days.
    10.2.2 Schedule of sampling
      10.2.2.1 Day of oocyte retrieval (day 12–16)
  10.3 Seminal Plasma (SE)
    10.3.1. Preparation
      10.3.1.1 Collect ejaculate in sterile 100-ml beaker right before oocyte retrieval.
      10.3.1.2 Note time of sampling.
      10.3.1.3 Print label with name and sample ID of the patient and label a 15-ml centrifuge tube.
      10.3.1.4 Transfer ejaculate into a 15-ml centrifuge tube with a sterile transfer pipette.
      10.3.1.5 Centrifuge 20 min at 3000 rpm.
      10.3.1.6 Gently take off supernatant (1–3 ml) with a 1-ml syringe.
      10.3.1.7 Transfer to a second 15-ml centrifuge tube.
      10.3.1.8 Centrifuge 20 min at 3000 rpm.
      10.3.1.9 Collect supernatant in a third 15-ml centrifuge tube (labeled).
      10.3.1.10 Put a drop of the supernatant on an object slide and check for successful removal of sperm cells with phase contrast microscope (×40 objective).
      10.3.1.11 Add another centrifugation step (20 min, 3000 rpm) if there are still sperm cells in the sample.
      10.3.1.12 Store tube at 4 °C until transfer to biobank.
      10.3.1.13 Right before transport, put samples in a polystyrene rack and deliver the box to a haulage service (4 °C).
      10.3.1.14 The haulage service assures a save transport to biobank under a consistent refrigerated temperature.
      10.3.1.15 Samples have to reach biobank within 24 h after sampling.
      10.3.1.16 Biobank assures validity of obtained SE samples by checking the respective barcode.
      10.3.1.17 Open SE samples manually and place into a Hamilton sample carrier.
      10.3.1.18 Fully automated Hamilton pipetting robot ML-STAR splits obtained serum samples into 1-ml aliquots.
      10.3.1.19 Store SE samples at −80 °C in storage systems of biobank for long-term storage.
    10.3.2. Schedule of sampling
      10.3.2.1 Day of oocyte retrieval (day 12–16)
  10.4 Embryo culture supernatant (SU) and control supernatant (SUC)
    10.4.1. Preparation
      10.4.1.1 The collection of SU and SUC is performed by embryologists or authorized staff.
      10.4.1.2 After end of the embryonal culture (=after embryonal transfer and/or embryo cryoconservation) the culture supernatant (SU) and control (SUC) will be collected immediately (refer to 10.2.1.4 for IVF preparation).
      10.4.1.3 Note time of sampling.
      10.4.1.4 Place embryo slide culture dish in a sterile workbench under a stereomicroscope.
      10.4.1.5 Aspirate the mineral oil layer and gently absorb approx. 20-μl SU with a pipette tip (0.1–10 μl).
      10.4.1.6 Check culture supernatant for oil contaminations under microscope and in case of contamination, aspirate remaining oil.
      10.4.1.7 Print a label with name and sample ID of the patient and label a 1.5 ml Eppendorf tube.
      10.4.1.8 Transfer SU in a 1.5-ml Eppendorf tube.
      10.4.1.9 Change pipette tip and repeat the process with other embryo culture wells.
      10.4.1.10 Aspirate and transfer embryo culture control supernatant SUC (cultivated without embryo; see 10.2.1.4.9) to a 1.5-ml Eppendorf tube.
      10.4.1.11 Store tubes at 4 °C until transfer to biobank.
      10.4.1.12 Right before transport, put samples in a polystyrene rack and deliver the box to a haulage service.
      10.4.1.13 The haulage service assures a save transport to biobank under a consistent refrigerated temperature (4 °C).
      10.4.1.14 Samples have to reach biobank within 24 h after collection.
      10.4.1.15 Biobank Graz assures validity of obtained SU and SUC samples by checking the respective barcode.
      10.4.1.16 Open SU and SUC samples manually and place into a Hamilton sample carrier.
      10.4.1.17 Fully automated Hamilton pipetting robot ML-STAR transfers samples into storage tubes (lobe tubes).
      10.4.1.18 Store SU and SUC samples at −80 °C in storage systems of biobank for long-term storage.
    10.4.2. Schedule of sampling
      10.4.2.1 Day 17–19 (day of embryo transfer and/or embryo cryoconservation) after 3–5 days of embryo culture
11. Sample usage
  11.1 Obtained samples can be analyzed with a variety of techniques including proteomic and metabolomics techniques.

## Discussion

The Kinderwunsch Institut Schenk, Dobl, Austria, has started to collect and store samples of FF, SE, SR, SU, and SUC in 2013. To date, more than 9000 different samples have been collected and stored at Biobank Graz of the Medical University of Graz. All samples from women and men undergoing IVF treatment have been approved for sampling and storage. The high quality samples can be tracked and assured by accurately following the SOP guidelines. This SOP serves as a template for other institutions in the field of reproductive health research to unify specimen collection procedures within the process of IVF.

One major advantage of the present SOP is to routinely collect samples, which would usually be designated as waste. Hence, a comprehensive dataset can be obtained prospectively for any type of retrospective study subsequently. The dataset allows linkage of specific sample types to clinical characteristics of the patients such as body mass index (BMI), smoking habit, age, and type of ovarian stimulation as well as pregnancy success and outcome.

Within the obtained dataset, follicular fluid is a parameter with increasing importance for IVF treatment. As plasma ultrafiltrate, follicular fluid comprises signaling mediators, mostly peptide hormones [13, 14], responsible for communication between granulosa cells, cumulus complex, and oocyte and somatic compartment [15–18]. This signaling is mandatory for maintenance of oocyte integrity. In addition to the ultrafiltrate, proteins can pass the follicular barrier, depending on molecular weight and charge characteristics [19]. The fluid components within the follicular fluid are potential sources for biomarkers defining oocyte health. Different ways of sampling and storage of follicular fluid are critical issues between datasets and the comparability remains uncertain. The current SOP addresses these issues and provides standardized sample consistency, accuracy, and quality.

By now, the collection of samples obtained from the Kinderwunsch Institut Schenk is unique in Austria and serves as the major source for reproductive health research in Austria. To the best of our knowledge, no laboratory facility collects follicular fluid per oocyte and stores this comprehensive dataset of FF, SE, SR, SU, and SUC of patients receiving IVF treatment in a biobank. The idea of storage of all these materials refers to basic research in IVF and was invented to gain knowledge of basic procedures in order to improve clinical results and pregnancy outcome. So far, the samples collected with the present SOP have already been used for scientific purposes. Quantification of protein carbonyl groups as oxidative stress parameter has been successfully analyzed in seminal plasma, embryonal culture supernatant, and follicular fluid [20]. Since the SOP was developed quite recently, there is lots of unexploited potential for scientific questions.

The cooperation with Biobank Graz of the Medical University of Graz enables investigators from all over the world with straightforward access to stored samples. We provide this SOP for a standardized quality management to assure good scientific practice and high quality of samples within different institutions. Hence, comparability and sample reliability can be guaranteed for state of the art molecular analysis in future studies in the field of reproductive health research. By following the SOP, sample handling and storage will be standardized and research comparability in institutions implementing the same procedures can be assured. This is a big step forward towards sound and reliable research.
